# Palliative care training: a national study of internal medicine residency program directors in the United Arab Emirates

**DOI:** 10.1186/s12904-022-00935-2

**Published:** 2022-04-01

**Authors:** Halah Ibrahim, Thana Harhara

**Affiliations:** 1 Khalifa University College of Medicine and Health Sciences, Abu Dhabi, United Arab Emirates; 2 Department of Medicine, Sheikh Khalifa Medical City, Abu Dhabi, United Arab Emirates

**Keywords:** Palliative care, End-of-life care, Graduate medical education, Residency, Internal medicine residents, United Arab Emirates, Program directors

## Abstract

**Background:**

Internal medicine residents are responsible for providing much of the direct care for palliative and terminally ill patients in teaching hospitals in the United Arab Emirates (UAE). To date, little systematic information is available on the prevalence of palliative care (PC) programs or faculty in UAE academic hospitals, or on the nature of PC education in internal medicine residency programs in the country.

**Methods:**

Semi-structured interviews were conducted with program directors of all 7 internal medicine residency programs in the UAE. Qualitative content analysis was conducted to identify recurring themes.

**Results:**

All program directors agreed that PC knowledge and skills are an essential component of training for internal medicine residents, but have had variable success in implementing the components. Three themes emerged, namely lack of structured PC training, perceptions of resident preparedness, and barriers to implementing a PC curriculum.

**Conclusion:**

Internal medicine residency programs in the UAE currently lack structured, mandatory PC curricula and have limited opportunities for formal teaching and assessment of PC knowledge and skills. The planned development of comprehensive oncology and palliative care centers and ongoing curricular reform in teaching hospitals in the country will provide important opportunities to train a cadre of competent health professionals to provide high quality palliative and end-of-life care to UAE patients and their families.

**Supplementary Information:**

The online version contains supplementary material available at 10.1186/s12904-022-00935-2.

## Background

Palliative medicine is receiving increased attention worldwide. The World Health Organization (WHO) defines palliative care (PC) as specialized care to improve the quality of life, through early identification and symptom relief, for patients and families dealing with serious illness [1]. Early referral to palliative care services has been shown to decrease symptom burden, and improve the quality of life for patients and caregivers, as well decrease healthcare costs [2]. As the world’s population ages and the prevalence of chronic, non-communicable diseases increases, the need for PC services is also rising, as is the demand for healthcare professionals who have the requisite knowledge and skills to care for this patient population [3]. Worldwide, there are not enough PC specialists to meet the current or projected need [4]. In fact, PC and the relief of suffering are considered among the most neglected dimensions of health care provision and health education globally [5].

The WHO cites the lack of trained health professionals as the major barrier in accessing PC services [6]. Several recent studies have further shown that the expansion of PC services requires the integration of PC teaching into medical school curricula and residency training programs [7–9]. A European study found that the lack of PC training, particularly in medical schools, was considered the greatest barrier to advancement of PC in the region [10]. Researchers in Israel noted expansion of PC teaching in the country’s medical schools in response to calls for greater access to PC services [9]. However, there is a large body of literature, spanning multiple countries and several decades, describing lack of knowledge, confidence, and preparedness in dealing with palliative and end-of-life issues by medical trainees and practicing health professionals [8, 11–13].

In the United Arab Emirates (UAE), palliative medicine is in its infancy. To date, only four hospitals in the country offer dedicated palliative services, and most exclusively treat patients with cancer diagnoses [14]. Additional palliative care programs are currently in development, alongside comprehensive cancer facilities in several hospitals. Yet, improving palliative and end-of-life care for UAE patients and their families will require not only this planned expansion of facilities and services, but also the proper training of all health professionals to provide generalist PC. A recent nationwide study of deans in all UAE medical schools noted deficiencies in undergraduate palliative medicine education, with limited clinical exposure [15]. To date, little systematic information is available on the prevalence of specialized palliative medicine faculty in UAE academic hospitals, or on the nature of PC education in residency training programs in the country. It is particularly important to understand the training in palliative and end-of-life care provided to internal medicine (IM) residents, as these trainees provide much of the care for seriously ill and dying patients in the hospital setting in the UAE. Therefore, this study was conducted to assess the current status of PC education, focusing on barriers and enablers to successful implementation of a PC and end-of-life curriculum, in all IM residency programs in the UAE.

## Methods

### Setting and participants

Internal medicine residency training in the UAE is structured, competency based training of four years duration. Each residency program is led by one program director (PD). We conducted a purposive sampling of PDs of all Arab Board accredited IM residency programs in the country. The PD is often a senior physician member of the hospital’s teaching faculty and is accountable for all aspects of resident education, including teaching, supervision, and assessment of the residents, as well as curriculum development, policy implementation and program administration. At the time of this study, there were 7 IM residency programs nation-wide, each led by a PD. Two of the training programs have since merged into one larger program. The Sheikh Khalifa Medical City institutional review board in Abu Dhabi, UAE reviewed and approved the study protocol [RS-564].

### Data collection

We followed the COnsolidated criteria for REporting Qualitative research (COREQ) for collecting and reporting our data [16]. One of the authors (TH), who completed formal training in medical education and palliative care, developed the initial semi-structured interview guide (Attachment 1). Four faculty members, who are involved in postgraduate medical education in the UAE, piloted the questionnaire for clarity and comprehension; they did not participate in the final study. The questions aimed to understand the depth and breadth of PC and end-of-life education delivered in the residency programs, as well as resident clinical exposure to PC patients. Questions included basic demographic information (rotations offered) and open-ended questions regarding details of PC teaching offered, including mandatory or elective rotations, time frames, content offered, teaching methods, and assessments. In addition, we sought information on the curriculum’s strengths and weaknesses, as well as planned curricular or policy changes related to PC education. We also aimed to better understand potential challenges the PDs faced in teaching palliative medicine to IM residents. We performed data collection and analysis concurrently, and adjusted the interview guide as new topics arose.

Both authors conducted semi-structured interviews with the PDs of all IM residency programs in the UAE. PDs were identified through institutional websites or by personal contacts. If the PD was unavailable, we interviewed a designee who was knowledgeable of all aspects of the residency curriculum. All respondents signed an informed consent sheet that explained the study purpose, risks and benefits, and confidentiality. Participation was voluntary, without incentives offered. We contacted each PD by email and provided details of the study and an invitation to participate. Interviews were then scheduled at the PDs’ convenience. The interviews were conducted in-person or over the phone between November 2019 and February 2021. We scheduled the phone interviews in advance so that they would be conducted in locations that offered privacy and minimal interruptions. The interviews were conducted in English, lasted 25–35 min and were audio recorded after obtaining participant consent. No additional notes were taken. Recordings were transcribed by professional transcriptionists, de-identified and checked for accuracy.

One of the authors (HI) independently performed a line-by-line review of the transcripts, generating initial codes. Qualitative content analysis was conducted, whereby both authors collaborated to identify similar concepts in the codes to group them into themes [17]. These themes were then applied to all transcripts [17]. All data coding and analyses were performed manually. The main findings are presented, with representative quotes.

## Results

All PDs from the 7 IM residency programs in the UAE, or their designees, participated in the study. Specifically, we interviewed 6 PDs and 1 faculty member, who was involved in the program’s curriculum development. Residency program characteristics are listed in Table 1. Three major themes emerged from the interviews, namely lack of structured PC training, PD perceptions of resident preparedness, and barriers to implementing a PC curriculum. The themes are discussed below, with quotes from the PDs to evidence our findings.

**Table 1:** Characteristics of UAE internal medicine residency programs

Emirate	Sponsoring Hospital	Type of Hospital	Palliative Care Service Available	Palliative Care Faculty on Staff	Year of Residency Program Development	Current Total Number of Residents
Abu Dhabi	Al Ain Hospital	Gov	No	No	2013	(Merged with Tawam Hospital in 2021)
	Cleveland Clinic Abu Dhabi	Private	No	Yes	2018	12
	Sheikh Khalifa Medical City	Gov	No	Yes	2006	46
	Sheikh Shakhbout Medical City (formerly Mafraq Hospital program)	Gov	No	No	2020 (Mafraq 2008)	27
	Tawam Hospital	Gov	Yes	Yes	2005	60 (Al Ain and Tawam combined)
	Zayed Military Hospital	Military	No	No	2009	21
Dubai	Dubai Hospital	Gov	No	No	2005	52

*Gov* Government

### Lack of structured palliative care training

The PDs agreed that PC knowledge and skills are an essential component of training for IM residents, but have had variable success in implementing the components of palliative medicine. Only one residency program offers a structured PC curriculum, though several are currently in the process of developing curricula. Only one program mandates a focused PC clinical rotation; the allotted time is for 2 weeks annually, and exposure is limited to patients with cancer diagnoses. One additional program offers an elective rotation in PC, but the PD reported that residents do not select this elective. She explained:

“Residents have this misconception that palliative care is about people who are dying. It could be depressing, because all they see are cancer patients. They don’t have the awareness that they need to be exposed to this sub-specialty.” [PD#1]

In all of the residency programs, PC exposure is embedded into other rotations, namely oncology, intensive care, general internal medicine, and geriatrics. One PD described:

“Dealing with or caring for a terminally ill patients is not clearly stated in the curriculum, but it goes with each rotation… Like for example, they’re going to have patients with end-stage COPD [chronic obstructive pulmonary disease] or we have someone with end-stage heart failure or renal failure. This is all palliative care, but there is not a specific separate palliative care rotation.” [PD#2]

#### Content of palliative care education

Three of the residency programs include PC didactic sessions in the curriculum, though often delivered by non-palliative care specialists. The most common topics covered include core principles of palliative care, pain management and end-of-life communication, including goals of care discussion. Other than pain issues, symptom management at end of life is rarely covered in formal didactic sessions, but is variably taught informally by non-palliative care specialists during patient care encounters. One PD explained:

“There are usually not sessions in the academic day- it’s usually discussions during rounds. We don’t usually say we are talking about a palliative care topic. But we’d have a case and the residents get asked how would you manage this patient? What medications would you give to relieve his symptoms? How would you break the news to the patient and his family that this is end-of-life care?” [PD#3]

#### Assessment of palliative care knowledge and skills

There is infrequent assessment of PC knowledge or skills. One PD stated:

“On the in-training exam from the US, I’m sure there is a question about palliative care. But we do not include palliative care questions on our internal exams.” [PD#1]

A few of the programs conduct workshops or annual objective structured clinical examinations (OSCEs), which focus on breaking bad news-type communication skills. Assessment of trainee serious illness communication skills occurs primarily during these ‘breaking bad news’ sections on OSCEs or workshops. The PDs admitted that residents are rarely, if ever, observed or provided feedback on their end-of-life patient care or communication.

### Perceptions of resident preparedness

All PDs acknowledge that the residents provide direct care to palliative and terminal patients, but feel that they have gaps in theoretical knowledge and skills. One PD explained:

“Clinical management-wise, I think the residents’ experience is developing and improving, but certainly they’re not competent to provide independent care to palliative patients, even the seniors.” [PD#4]

The PDs also felt that residents needed more training in serious illness communication skills, particularly initiating goals of care conversations. One PD described:

“We will be discussing communication with the family whenever we see any terminal or dying patients, but again they [the residents] don’t have the formal training. So it is always up to the consultant [attending physician] to break the bad news.” [PD#5]

### Barriers to implementing a palliative care curriculum

#### Lack of specialized palliative care faculty

All PDs felt that the dearth of palliative medicine expertise was the main barrier to implementing a PC curriculum. Only one of the academic hospitals has a palliative care unit. Three programs have faculty members who have specialized training and/or expertise in PC; though several programs expect the recruitment of PC specialists within the next few years, as their hospitals are in the process of expanding their oncology services and developing specialized oncology centers with dedicated PC units. Programs are unable to offer an interprofessional approach to palliative medicine. None of the programs have included other disciplines, such as social workers, faith-based or spiritual leaders, or psychologists, in their PC teaching, primarily because their hospitals currently lack this expertise. One PD elaborated:

“We don’t have enough expertise in the system to deal with palliative patients and palliative medicine education. We don’t have the team that takes care of all the aspects. Because if you want to teach this, you need the whole team there, like social worker, case managers, psychologist, counselor, the nurses, the spiritual leader. Everyone needs to be on board, together, to make it easier for us to deliver this kind of education.” [PD#6]

#### Cultural resistance

Several PDs reported a lack of public awareness and cultural resistance to initiating PC services. One PD described:

“There is an underestimation of this important specialty. People here think palliative care means patients will die. Families don’t have the awareness or education to know the benefits of palliative care.” [PD#5]

#### Lack of formal hospital end-of-life policies

Several PDs cited the lack of formal hospital policies regarding end-of-life care to be a barrier to the implementation of PC education. One PD explained:

“A DNR [do not resuscitate] policy has been implemented, but we still don’t have a law to suggest who’s the surrogate or the power of attorney, for example. You know, all these things are not there, but you need them if you want to do it right.” [PD#7]

#### Inadequate support for residents

Debriefing rarely occurs after patient deaths, and none of the programs offer residents formal venues for psychological support.

## Discussion

This study provides a snapshot of the structure and content of palliative care education in the hospitals that train IM residents in the UAE. Overall, IM PDs agreed that proficiency in palliative and end-of-life care was essential, but faced challenges in implementing its components. PDs recognized that IM residents frequently provide direct care to palliative and terminal patients in the hospital. Yet, their training in PC was limited in scope and duration and lacked formalized feedback, revealing inconsistencies and fragmentation across experiences. Training deficits in PC skills are not unique to UAE IM residents. Our findings are consistent with multiple studies across many countries, in which residents reported inadequate training in palliative medicine and desired additional clinical exposure to PC services [11–13, 18]. Other studies have also noted that many residents are rarely observed conducting serious illness discussions or provided feedback from faculty on these communication skills [19].

The major curricular challenge reported by the PDs was the dearth of specialized PC faculty in most of the hospitals. Also, the programs lacked the human resources to provide a multidisciplinary team model of holistic palliative care. Social workers, PC nurses, faith-based professionals, and bereavement counselors were rarely involved in the PC education of the IM residents, depriving the trainees of critical perspectives in palliative care. Studies of PC education in other countries have had similar conclusions [7, 9, 10].

It is notable that several PDs cited a lack of formal institutional policies regarding end-of-life care, particularly regarding “do not resuscitate” regulations, as a barrier to implementing a PC curriculum. The UAE established a national policy in 2016 to allow natural death in patients suffering from a terminal illness [20]. Lack of awareness or inconsistent implementation of end-of-life policies can be an impediment to the provision of PC services.

Finally, none of the programs offered formal debriefing sessions or mechanisms for residents to discuss the emotions inherent in caring for dying patients. A multi-institutional study in Canada reported that almost half of the residents surveyed reported feelings of guilt or failure after a patient’s death [13]. The literature suggests that all residents should receive structured debriefing that explores effective coping strategies, as well as training in personal reflection on their experiences with patient death [14]. There are challenges expected in facilitating self-reflection in medical trainees [21]. The literature provides several tools to help overcome these barriers [21–23]. A recently published EAPC white paper on multidisciplinary education for spiritual care in PC provides evidence-based strategies, including Schwartz Rounds [22], a forum that allows for a safe space for healthcare professionals to share stories regarding the emotional and social aspects of patient care, and Circle of Trust [23], which allows the medical team to reflect on their spiritual care practices.

As education is a central measure of PC status worldwide, the lack of standardized PC education in the country’s residency programs stands as a barrier to the advancement of PC services in the UAE. Curricular changes are necessary to improve PC and end-of-life training for our IM residents. Studies have shown that comprehensive educational interventions or dedicated palliative care rotations can improve trainee proficiency [7, 9, 24]. Several educational interventions have already been developed in other countries and have demonstrated success. For example, the EDUPALL project represents a blended, standardized undergraduate PC curriculum available to countries worldwide [25]. These interventions can be locally adapted to improve physician education in the UAE. Research has also shown that PC education should become a mandatory subject in medical schools and residency programs [9]. We agree that a structured PC curriculum, with both didactic and clinical components, should be a mandatory part of all IM residency programs and should be formally adopted by the Emirati Board, which is currently in development. Faculty development is also necessary for healthcare providers at all levels to improve their knowledge and skills of core PC principles. For example, a weeklong multi-professional course, delivered in the Ukraine, showed improved participant self-assessment of knowledge and facilitated networking and team building [26].

Our study has several limitations. First, a small number of PDs were interviewed, but they represent all of the accredited IM residency programs in the UAE at the time of the study, and we believe our findings represent the current PC curriculum being taught to IM residents in the country’s teaching hospitals. The PDs may have presented their programs in a favorable light, thereby, overestimating the presence of meaningful PC training. We only report the presence of PC training, but are unable to assess the quality of the education. Also, only IM programs were included. We believe that proficiency in PC should be a goal for graduates of all residency programs. Further, the resident, patient and caregiver perspectives are missing. Finally, investigator bias must be considered. As both investigators are medical educators with an interest in palliative medicine, we were mindful that our previous experiences and assumptions could have impacted our data analysis. To minimize bias, we frequently questioned and discussed each other’s conclusions. Future studies are needed to assess the impact of palliative care training on the IM residents and their care of palliative patients and their families. Despite these limitations, we are hopeful that the findings from this study will inform curriculum developers and educational policy makers in UAE residency programs.

## Conclusion

Palliative care is an essential component of residency training. Yet, most IM residency programs in the UAE currently lack structured palliative medicine curricula, and provide limited formal teaching and assessment of trainee knowledge and skills in palliative and end-of-life care. Curricular reform will be necessary to train a cadre of competent health professionals, who can provide high quality palliative and end-of-life care to UAE patients and their families. Future studies should include resident perspectives on their experiences and learning needs.

## Supplementary Information

**Additional file 1.** 

## Data Availability

Data can be provided by corresponding author upon reasonable request.

## Abbreviations

IM: Internal medicine
PC: Palliative care
PD: Program directors
UAE: United Arab Emirates
