# Supplementary material for: Palliative care training: a national study of internal medicine residency program directors in the United Arab Emirates
Source: BMC Palliat Care. 2022 Apr 1;21:44. doi: 10.1186/s12904-022-00935-2 (PMC8973813; doi:10.1186/s12904-022-00935-2)
Supplement: Supplementary file 1 — Additional file 1. [file 12904_2022_935_MOESM1_ESM.docx]

**
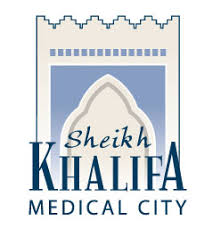
**

**Palliative Care Curriculum in Internal Medicine Residency Programs in the UAE**

**Interview Guide:**

For the purpose of our conversation, **palliative care** is defined as an approach that improves the quality of life of patients and their families facing problems associated with life-threatening illness, through the prevention and relief of suffering (WHO).

**End of Life care** is defined as care for people in decline who are deemed to be terminal or dying in the foreseeable future (CIHI).

**Palliative Care Teaching and Resources**

1. Are your residents given any formal teaching on palliative care and end-of-life care?

Why? Why not?

Who/what led to the introduction of the teaching?

1. If yes, what format do you use for palliative care education for your residents?

How are they structured?

1. What palliative care topics are covered?

Are they covered explicitly as part of an End-of-Life (EOL) and palliative care curriculum?

Do you have any topics that are related to palliative care but covered under different lectures for example geriatrics, anesthesia, ethics, or communication skills? Can you give me examples?

1. Does your program have any faculty members who are trained in palliative medicine?

**Palliative Care experience**

1. Are your residents involved in taking care of terminally ill patients or patients at EOL?

How much exposure do you think your residents have to such patients?

How do your residents deal with EOL patients?

1. Do your residents participate in a mandatory rotation in palliative care?

Why? Why not?

If they do, how long and where is the rotation?

1. Do your residents participate in an elective rotation in palliative care?

Why? Why not?

If they do, how long and where is the rotation?

What percentage of your residents choose palliative care electives?

1. In what clinical settings does palliative care teaching take place at your institution?

**Evaluation**

1. Do you evaluate your residents’ knowledge in palliative care?

How?

**Learning Needs and barriers**

1. Is it important to your institution that residents learn about how to provide care for dying patients?

Why?

1. Would you be supportive of integrating end-of-life care into your curriculum?

Why?

How would you integrate it?

1. In your opinion, what are potential barriers to teaching end-of-life care in postgraduate medical education?

**Thank you for your time. Do you have anything else you would like to share?**
